# Catatonic Symptoms in Egyptian Autistic Specrum Disorder Children

**DOI:** 10.1192/j.eurpsy.2026.11125

**Published:** 2026-07-31

**Authors:** A. N. R. B. Saad

**Affiliations:** Neuropsychiatry, Menoufia, Menoufia, Egypt

## Abstract

**Introduction:**

Autistic spectrum disorder (ASD) is a complex neurodevelopmental disorder with a broad of symptoms including social communication deficits, repetitive behaviours, and restricted interest. Catatonia on the other hand is neuropsychiatric syndrome characterized by motor abnormalities, behaviour disturbances and altered consciousness. They share a lot of symptoms and neurobiological basis so the diagnosis and early intervention is challenging

**Objectives:**

I aimed to assess catatonic symptoms in a sample of Egyptian ASD children

**Methods:**

511 autistic children (250 male and 261 female), aged from (3-13 years) were recruited from outpatient clinic of Menoufia university hospital from April 2020 to September 2020 with exclusion of severe intellectual disabled children, children with organic brain lesions or epilepsy and uncontrolled medical illnesses and 511. Semi structured interview used to collect information from parents and examination of children including mental assessment, neurological assessment, IQ test, childhood autistic rating scale 2, Brush- Franscis Catatonia Rating Scale, and Electroencephalography.

**Results:**

Out pf the patients I found that 32 ASD patients (6.2%) have met the criteria of catatonic symptoms, and there were positive correlation with severity of autistic symptoms, repetitive symptom domain and abnormal EEG with catatonia.

**Conclusions:**

Patients with autism should be explored carefully to symptoms of catatonia especially high risk children of low IQ, repetitive movements domain and abnormal EEG.

**Disclosure of Interest:**

None Declared

